# Google Earth elevation data extraction and accuracy assessment for transportation applications

**DOI:** 10.1371/journal.pone.0175756

**Published:** 2017-04-26

**Authors:** Yinsong Wang, Yajie Zou, Kristian Henrickson, Yinhai Wang, Jinjun Tang, Byung-Jung Park

**Affiliations:** 1 Shanghai International Automobile City (Group) Co., Ltd, Shanghai, P.R.China; 2 Key Laboratory of Road and Traffic Engineering of the Ministry of Education, Tongji University, Shanghai, P.R.China; 3 Department of Civil Engineering, University of Washington, Seattle, Washington, United States of America; 4 School of Traffic & Transportation Engineering, Central South University, Changsha, China; 5 Department of Transportation Engineering, Myongji University, Seoul, Korea; Beihang University, CHINA

## Abstract

Roadway elevation data is critical for a variety of transportation analyses. However, it has been challenging to obtain such data and most roadway GIS databases do not have them. This paper intends to address this need by proposing a method to extract roadway elevation data from Google Earth (GE) for transportation applications. A comprehensive accuracy assessment of the GE-extracted elevation data is conducted for the area of conterminous USA. The GE elevation data was compared with the ground truth data from nationwide GPS benchmarks and roadway monuments from six states in the conterminous USA. This study also compares the GE elevation data with the elevation raster data from the U.S. Geological Survey National Elevation Dataset (USGS NED), which is a widely used data source for extracting roadway elevation. Mean absolute error (MAE) and root mean squared error (RMSE) are used to assess the accuracy and the test results show MAE, RMSE and standard deviation of GE roadway elevation error are 1.32 meters, 2.27 meters and 2.27 meters, respectively. Finally, the proposed extraction method was implemented and validated for the following three scenarios: (1) extracting roadway elevation differentiating by directions, (2) multi-layered roadway recognition in freeway segment and (3) slope segmentation and grade calculation in freeway segment. The methodology validation results indicate that the proposed extraction method can locate the extracting route accurately, recognize multi-layered roadway section, and segment the extracted route by grade automatically. Overall, it is found that the high accuracy elevation data available from GE provide a reliable data source for various transportation applications.

## Introduction

Roadway elevation data play a critical role in a wide range of transportation analysis and design applications including roadway geometric design, infrastructure construction, safety analysis, fuel consumption estimation, highway capacity analysis, and emergency evacuation planning [1–3]. Previous research has shown that vehicle performance and fuel efficiency are significantly affected by roadway elevation changes. The Highway Capacity Manual (HCM) 2010, for example, assigns values of heavy vehicle to passenger car equivalency factors based on grade changes [4]. Likewise, degradation of vehicle performance and sight distance at vertical alignments are often causes of recurrent congestion and vehicle collisions [5–7]. Previous work has also identified non-linear relationships between roadway grade and fuel economy. Boriboonsomsin and Barth [8] showed that the optimal speed in terms of fuel efficiency changes with grade. Such findings on the relationship between roadway grade and safety, fuel consumption, and network performance indicate that the availability and quality of roadway elevation and grade data will be a critical consideration in the development of a next generation “green” highway design strategy that integrates life cycle maintenance, operation, safety, and environmental cost in the planning stage.

Traditional roadway Geographic Information System (GIS) data, however, contain only two dimensional geo-coordinates, missing the elevation information in most cases. The earliest method for collecting elevation data was to manually survey and draw isolines of elevation. Over the past few decades, new data processing methods and data collection, storage, query, and visualization technologies have significantly increased the availability and accessibility of elevation data. Currently available datasets include the global 30 arc-second elevation (GTOPO30) dataset [9], elevation dataset from Shuttle Radar Topography Mission (SRTM) [10], National Elevation Dataset from the U.S. Geological Survey (USGS NED) [11], Global Digital Elevation Model (GDEM) [12], and Light Detection and Ranging (LIDAR) [13] elevation datasets. Presently, the quality of readily available elevation data varies by source and acquisition technology. GTOPO30 is based on several different source datasets and has variable absolute vertical accuracy. The usefulness of GTOPO30 for deriving the roadway elevation is questionable because of its low resolution and the inherent vertical uncertainty of the multiple elevation data sources. The elevation data from SRTM are available at a 3 arc-second (about 90 meter) resolution with an 80% global coverage. The USGS NED data are available at a grid spacing of 1 arc-second (about 30 meters) for the conterminous USA, and at 1/3 and 1/9 arc-second grids (approximately 10 and 3 meters, respectively) for parts of the nation. Most of the USGS NED data for Alaska are available at a 2-arc-second (about 60 meters) grid spacing because only lower resolution source data exist there. The GDEM data are the most widely covered elevation data source (from 83°N to 83°S, covering about 99% the globe) with a grid resolution of 30 meters. A vertical accuracy study found the root mean square error (RMSE) of GDEM data is 8.68 meters when compared against 18,000 geodetic control points in the USA [14]. LIDAR-derived elevation data are available for some coastal states and inland states at a resolution of 1/9 arc-second (about 3 meters).

Although many sources of elevation data are available at very low costs, methods to acquire public roadway elevation data on a large-scale based on these resources are currently lacking. With more than 200 million downloads since its release in June 2005 [15], Google Earth (GE) has recently been recognized for its potential to significantly improve the visualization and dissemination of scientific data [16–18]. The elevation of any points, including the multilayered bridges in some metropolitan areas, can be acquired with GE or its Application Programming Interface (API). Up to this point, Google has been unwilling to release detailed information regarding the accuracy of the archive, though some previous research work has addressed this issue on a limited scale. For example, Potere [15] evaluated the horizontal positional accuracy of GE’s imagery archive. Benker et al. [19] tested the horizontal and vertical positional accuracy of the GE terrain model in the Big Bend region, Texas, USA. This study intends to conduct a comprehensive assessment of GE elevation accuracy in the area of conterminous USA to examine whether GE elevation data is a valuable resource for transportation applications, and to develop the methods for bulk acquisition of public roadway elevation and grade data from GE.

The reminder of this paper is organized as follows: Section 2 describes the method for bulk data acquisition of roadway elevation and grade from GE, including the lower level road in multi-layered roadway sections. Section 3 investigates the GE elevation accuracy by comparing to the ground truth elevation data from GPS benchmarks and roadway monuments. Section 4 implements and validates the proposed roadway elevation extraction method. Section 5 provides the conclusion and recommendations for future research.

## Roadway elevation extraction method

### Method overview

**Fig 1:**
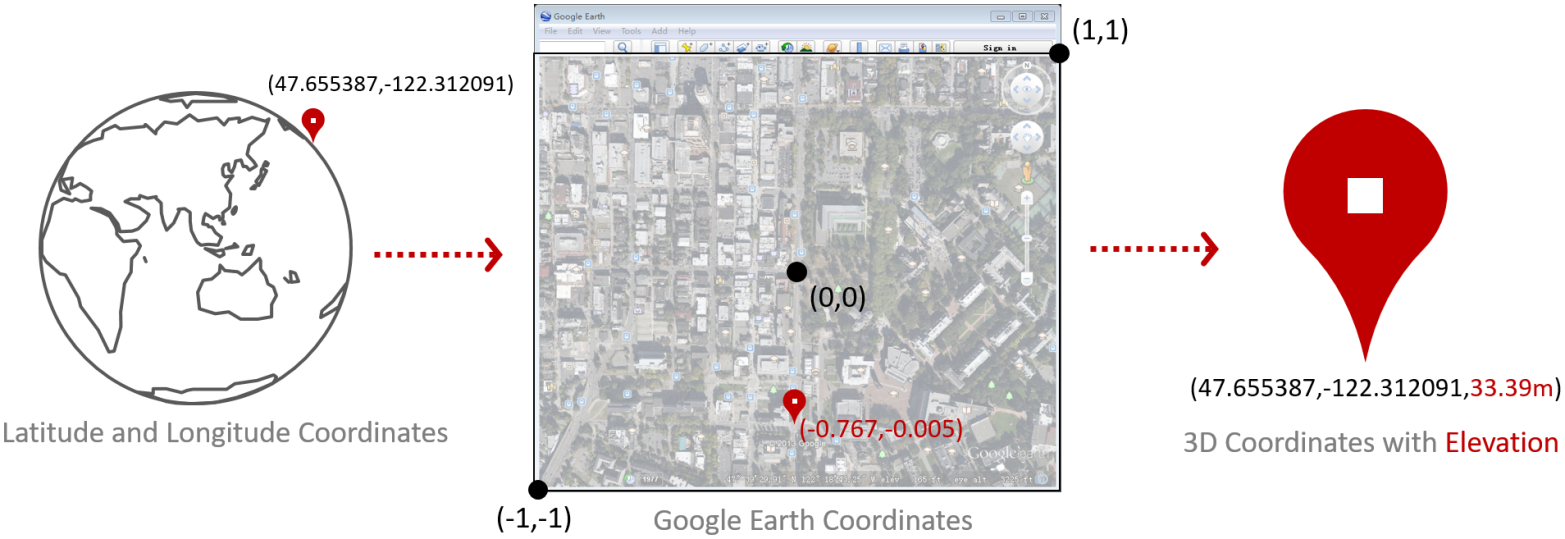
Sketch of Google Earth coordinates and coordinate transformation procedure.

To extract the roadway elevation and grade, a Google Earth Elevation Data Extraction System (GEEDES) was developed at the Smart Transportation Applications and Research Laboratory (STAR Lab) of the University of Washington using the GE API. The GE API only allows third-party applications to acquire the elevation at any points displayed on the GE application relative coordinates (see Fig 1). This is addressed in GEEDES with the following operation steps:

1. Determine GE viewbox parameters based on the start/end points and geometric information of the segment of interest;
2. Convert the latitude and longitude coordinates (widely used in roadway GIS) of sampling points into the GE form relative coordinates and extract the raw elevation data;
3. Multi-layered roadway recognition and data correction; and
4. Slope segmentation and grade calculation.

### GE viewbox parameters determination

The purpose of calculating GE viewbox parameters is to ensure the extracting routes are displayed in the GE form. The major viewbox parameters are the focus centre location *O*[*LO*_*fc*_,*LA*_*fc*_] in longitude and latitude format and the range of viewpoint *r*. The equations to calculate *O*[*LO*_*o*_,*LA*_*o*_] and *r* are:

$$\begin{array}{l} LO_{fc}=\frac{LO_{\text{min}}+LO_{\text{max}}}{2}\\ LA_{fc}=\frac{LA_{\text{min}}+LA_{\text{max}}}{2} \end{array}$$ (1)

$$r=\frac{\text{max}[f\left(LO_{\text{min}},LA_{\text{min}},LO_{\text{max}},LA_{\text{min}}\right)|f(LO_{\text{min}},LA_{\text{min}},LO_{\text{min}},LA_{\text{max}})]}{2\cdot \text{tan}(\theta /2)}$$ (2)

where *LO*_*fc*_ and *LA*_*fc*_ are the longitude and latitude of GE viewbox focus centre location *O*. *LO*_min_ and *LO*_max_ represent the minimum and maximum value of longitude of all the sampling points on the extracting route. *LA*_min_ and *LA*_max_ represent the minimum and maximum value of latitude of all the sampling points on the extracting route. *θ* indicates the camera angle of the GE viewbox, which is a fixed parameter predefined in GE. *f* is the function to calculate the distance between two points in latitude and longitude coordinates. Great-circle distance is a method known from spherical geometry [20] to calculate the distance between two points on a curved surface like the earth, but it has a significant drawback in that rounding error may be present when two points are located close to each other [21]. In this study, the Haversine-formula [20] has been used in the distance calculation function *f* to improve numerical stability:

$$f(LO_{i},LA_{i},LO_{j},LA_{j})=RE\times 2\text{arcsin}\left(\sqrt{{\text{sin}}^{2}\left(\frac{LA_{j}-LA_{i}}{2}\right)+\text{cos}(LA_{i})\text{cos}(LA_{j}){\text{sin}}^{2}\left(\frac{LO_{j}-LO_{i}}{2}\right)}\right)$$ (3)

where *LO*_*i*_, *LA*_*i*_, *LO*_*j*_ and *LA*_*j*_ are the longitude and latitude of any two points *i* and *j*, and *RE* is the radius of the earth.

### Coordinate transformation and raw elevation data extraction

By setting the GE viewbox parameters to *O*[*LO*_*fc*_,*LA*_*fc*_] and *r*, the entire extracting routes can be displayed in the GE form for extracting elevation. In the GE form box, the coordinate values of the lower-left corner, centre and upper-right corner are [–1,–1], [0,0] and [1,1] as shown in Fig 1. The locations of all the other points displayed in the GE form box are represented by the decimals between [–1,–1] to [1,1]. Considering the resolution of the GE viewbox, the displayed area in the GE form for extraction should not be very large and can be regarded as a plane. Therefore the location [*x*_*i*_,*y*_*i*_] of each sampling point *i* in the GE form relative coordinates can be converted from the longitude and latitude by Eqs 4 and 5:

$$x_{i}=\frac{f(LO_{i},LA_{fc},LO_{fc},LA_{fc})}{f(LO_{ur},LA_{fc},LO_{fc},LA_{fc})}\times \frac{LO_{i}-LO_{fc}}{\left|LO_{i}-LO_{fc}\right|}$$ (4)

$$y_{i}=\frac{f(LO_{fc},LA_{i},LO_{fc},LA_{fc})}{f(LO_{fc},LA_{ur},LO_{fc},LA_{fc})}\times \frac{LA_{i}-LA_{fc}}{\left|LA_{i}-LA_{fc}\right|}$$ (5)

where *LO*_*ur*_ and *LA*_*ur*_ represent the longitude and latitude of the upper-right corner in the GE form; *LO*_*i*_ and *LA*_*i*_ represent the longitude and latitude of each sampling point *i*; the elevation of point *i* then can be acquired by calling the “GetPointOnTerrainFromScreenCoords(*x*_*i*_, *y*_*i*_)” function in the GE API.

### Multi-layered roadway recognition and data correction

**Fig 2:**
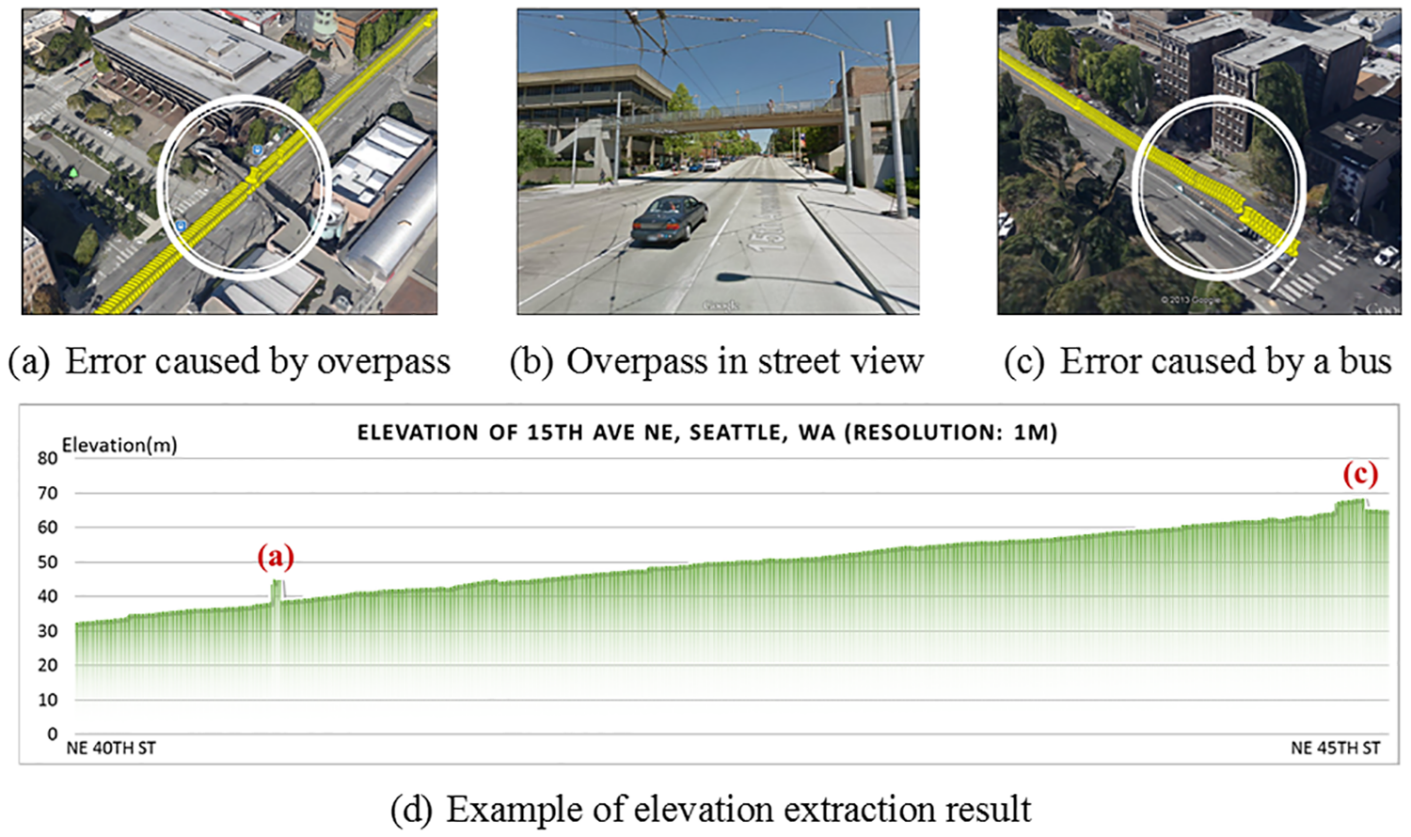
Example of exception data in roadway elevation extraction.

The method developed above can extract the elevation of any point by the given longitude and latitude. However, this method has a significant limitation that, as shown in Fig 2, only the top layer elevations can be measured, and the extracted roadway elevation is affected by overlapping infrastructure such as interchanges, multilayered roadways, or multideck bridges. Elevations of lower layer roadways, tunnels, and sheltered roadways cannot be directly measured using the aforementioned method. In addition to the overhead infrastructure, road surface and traffic conditions at the time of measurement may also cause errors as illustrated in Fig 2(c). It is necessary to further process the elevation data to eliminate errors induced by surface sheltering and estimate the elevation for the roadways that cannot be directly acquired.

In GEEDES, the sampling points of the extracting routes are evenly distributed along the routes at a predefined resolution. The prevailing feature of the errors caused by overlapping infrastructure is that the elevation along the lower layer route suddenly steps upward to the elevation of top layer in the overlapping segment, and then falls back to lower layer elevation at the end of overlap. Based on this characteristic, the following method has been developed to recognize the overlapping area: first, Δ*E*_*i*_ and *t*_*i*_ are defined in Eqs 6 and 7, describing the elevation difference and variation trend between each sampling point *i* and its previous one.

$$\Delta E_{i}=E_{i}-E_{i-1}$$ (6)

$$t_{i}=\left\{ \begin{array}{l} 1\begin{array}{cc} , & \Delta E_{i}>0 \end{array}\\ 0\begin{array}{cc} , & \Delta E_{i}=0 \end{array}\\ -1\begin{array}{cc} , & \Delta E_{i}<0 \end{array} \end{array}\right.$$ (7)

where *E*_*i*_ represents the elevation of sampling point *i* ordering by the distance from the starting point of extracting route. The method assumes that the infrastructure overlapping will not occur at the starting or ending section of the extracting route, and compares all the subsequent sampling points iteratively by the following procedure to find the overlapping areas and correct the elevation:

1. Check for new overlapping segment by elevation change of sampling point [*i*]:
   If (|Δ*E*_*i*_ | > *α*), then go to step 2); else proceed to step 1) for sampling point [*i+1*].
2. Check if previous segment [*i*] is on overlapping segment:
   If *F*_*m*_ = *false*, then go to step 3); else go to step 4).
3. Start new overlapping area beginning at segment [*i*]:
   Set *I*_*start*_ = *i*
   Set $\Delta e=\frac{\sum_{j=i-N}^{i}\frac{E_{i}-E_{j}}{i-j}}{n}$
   Set *F*_*m*_ = *true*
   Proceed to step 1) for sampling point [*i+1*].
4. Check whether end of overlapping segment is reached:
   If (*t*_*Istart*_ × *t*_*i*_ *< 0* and |Δ*e* × (*i—I*_*start*_) + *E*_*Istart*_—*E*_*i*_| < *β*), then go to step 5); else proceed to step 1) for sampling point [*i+1*].
5. Set index of overlapping ending point (*I*_*end*_): *I*_*end*_ = *i*,
   Set *F*_*m*_ = *false*
   Replace the elevation of sampling point between *I*_*start*_ and *I*_*end*_ by linear interpolation
   Proceed to step 1 for sampling point [*i+1*].

where *α* is the threshold to determine whether a jump occurs and *β* is the threshold to determine whether the overlapping segment has ended; *F*_*m*_ is a flag variable indicating whether the current point is within the overlapping segment; Δ*e* is the grade of the segment immediately before the overlapping segment; and *n* is number of sampling points utilized to calculate Δ*e*, which is determined by the sampling resolution.

### Slope segmentation and grade calculation

Slope length and roadway grade rather than the elevation of a single sampling point are useful for some transportation applications (i.e., fuel consumption calculation, eco-routing, etc.). Using the point elevation data obtained by the proposed extraction and correction method, this sub-section aims to develop an approach to segment the extracted route by recognizing roadway grade changes and calculate the slope length and grade. The basis for recognizing roadway grade changes in this study is similar to the method of multi-layered roadway recognition proposed in the previous sub-section. A rolling space interval is utilized to check whether a grade change occurs. The rolling space interval’s slope angle relative to the horizontal plane can be calculated by:

$$\varphi_{i}=\frac{\sum_{j=i}^{i+n}\text{arctan}(\frac{E_{i+\text{n}}-E_{j}}{(i+n-j)\times R})}{n},(-\frac{\pi }{2}<\varphi_{i}<\frac{\pi }{2})$$ (8)

$$\Delta \varphi_{start,i}=\varphi_{i}-\varphi_{start}$$ (9)

$$\Delta \varphi_{i,i-1}=\varphi_{i}-\varphi_{i-1}$$ (10)

where *φ*_*i*_ is the slope angle for the rolling space interval starting from sampling point *i* and *n* is the number of sampling points contained in the rolling space interval. *R* is the horizontal distance between two successive sampling points, which is determined by the sampling resolution. *φ*_*start*_ is the slope angle for the starting space interval of every new segment. Δ*φ*_*start*,*i*_ indicates the slope angle difference between rolling space interval *i* and the slope of the starting space interval. Δ*φ*_*i*,*i-1*_ indicates the slope angle difference between rolling space interval *i* and its previous one. The detailed procedure to segment the extracted route by grade is described below:

1. Check slope angle change of rolling space interval [*i*]:
   if (|Δ*φ*_*start*,*i*_ | > *γ* and *F*_*g*_ = *False*), then go to step 2); else check rolling space interval [*i+1*] in step1).
2. Set *F*_*g*_ = *True*, proceed to step 3) for rolling space interval [*i+1*].
3. Check for end of constant grade segment:
   If (|Δ*φ*_*i*,*i-1*_ | < *δ*), go to step 4); else proceed to step 3) for rolling space interval [*i+1*].
4. Begin new constant grade segment:
   Set *I*_*seg*,*k*_ = *i—1*
   Set *F*_*g*_ = *false*
   Set *φ*_*start*_ = *φ*_*i-1*,_
   Set *k = k +1*
   Proceed to step 1) for space interval [*i+1*].

where *k* is the constant grade segment index, *γ* is the threshold to determine whether an obvious slope angle change occurs, *δ* is the threshold to determine whether the rolling space interval enters a new segment with consistent grade, and *F*_*g*_ is a flag variable to indicate whether the rolling space interval enters variable slope section. For any segment between segmentation points *I*_*seg*,*k*_ and *I*_*seg*,*k+1*_, the length and grade can be calculated by Eq 11 through Eq 13:

$$L_{k}=\sum_{j=I_{seg,k}}^{I_{seg,k+1}-1}R\times \text{cos}(\text{arctan}(\frac{E_{j+1}-E_{j}}{R}))$$ (11)

$$g_{k}=\text{tan}(\frac{\sum_{j=I_{seg,k}}^{I_{seg,k+1}}\phi_{j}}{I_{seg,k+1}-I_{seg,k}})$$ (12)

$$\phi_{j}=\text{arctan}(\frac{E_{j}-E_{I_{seg,k}}}{(j-I_{seg,k})\times R}),(-\frac{\pi }{2}<\phi_{j}<\frac{\pi }{2})$$ (13)

where *L*_*k*_ is the length of segment *k*, *g*_*k*_ is the grade of segment *k*, and *ϕ*_*j*_ is the slope angle at sampling point *j* in segment *k*.

## GE elevation accuracy assessment

### Reference data

**Fig 3:**
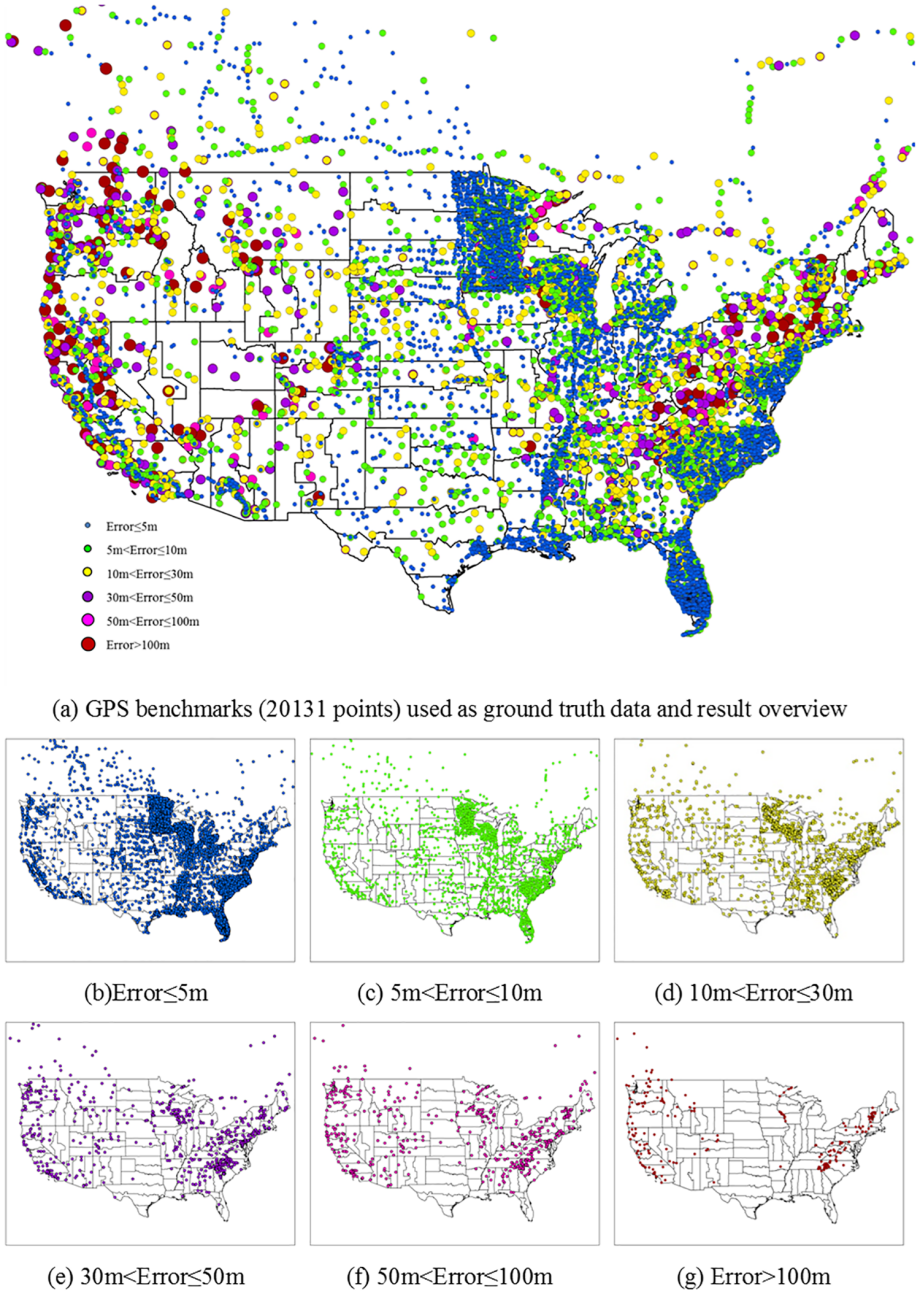
Sampling points distributions in the continuous USA on different GE elevation error levels.

With the extraction method proposed in this study, the applicability of the GE elevation for transportation applications depends on the accuracy of the original GE elevation data. This section aims to conduct a comprehensive assessment for the accuracy of GE’s original elevation data. Two datasets are utilized as ground truth data to examine the accuracy of the elevation data extracted from the GE. The first is the “GPS on Bench Markers” dataset of geodetic control points (http://www.ngs.noaa.gov/GEOID/GPSonBM09/) from the National Geodetic Survey (NGS). This set of points has millimetre to centimetre-level accuracies and covers the conterminous USA on a broad range of topographies (see Fig 3(a))[11]. To assess GE’s elevation accuracy for transportation use, roadway monuments directly adjacent to the roadways of interest are utilized as the second source of ground truth data. Roadway monuments data are provided by transportation agencies such as state DOTs, and also with the centimetre-level accuracy on both horizontal and vertical position. The GE elevation data used for comparison was extracted during April 15th~25th, 2014.

### Accuracy assessment indicators

In this study, common statistical indicators and statistical test such as Mean Absolute Error (MAE) standard deviation (Std. Dev), Root Mean Square Error (RMSE) and Mann-Whitney U Test are considered. Absolute vertical accuracy can also be expressed in terms of an error interval at a percentile, in many cases 95%, which is also referred to as “boundary of error interval at 95%” (BE 95). The accuracy assessment indicators used in this study can be calculated by Eq 14 through Eq 18:

$$MAE=\frac{\sum_{i=1}^{N}\left|{\hat{v}}_{i}-v_{i}\right|}{N}$$ (14)

$$u=\frac{\sum_{i=1}^{N}{\hat{v}}_{i}-v_{i}}{N}$$ (15)

$$Std.Dev.=\sqrt{\frac{\sum_{i=1}^{N}{\left({\hat{v}}_{i}-v_{i}-u\right)}^{2}}{N}}$$ (16)

$$RMSE=\sqrt{\frac{\sum_{i=1}^{N}{\left({\hat{v}}_{i}-v_{i}\right)}^{2}}{N-1}}$$ (17)

BE95=u±1.96×Std.Dev. (18)

where *N* is the number of observations, *v*_*i*_ is the ground truth elevation at point *i*, ${\hat{v}}_{i}$ is the measured elevation value, *u* is the mean error for all observations, and 1.96 is the value of the standard normal distribution z-statistic at cumulative probability = 97.5%.

### GE vs. GPS benchmarks

This study compared the elevation extracted from GE with 20131 GPS benchmarks in the conterminous USA. These benchmarks cover many types of land cover including developed city, forest, wetland, etc. The coverage of GPS benchmarks is shown in Fig 3(a). The spatial distribution of the different levels of GE elevation error measured relative to the GPS benchmark data are shown in Fig 3(a) through 3(g).

**Fig 4:**
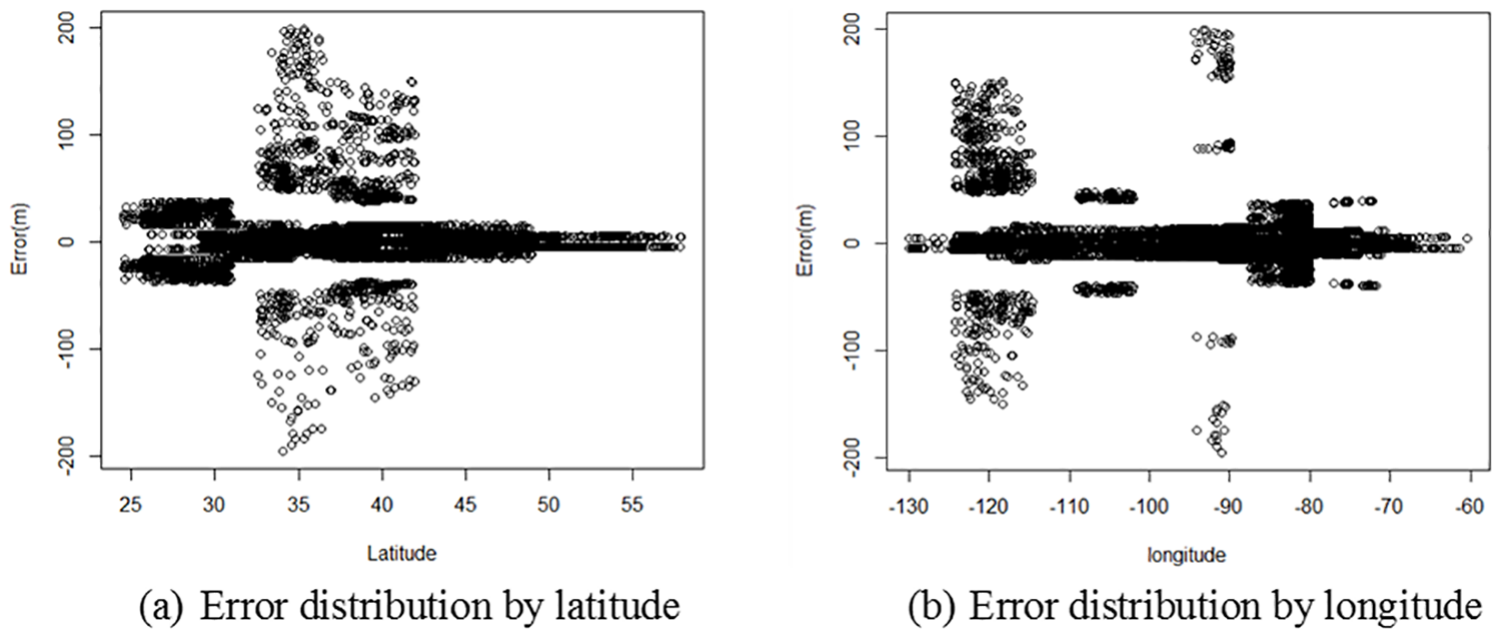
Error distributions by latitude and longitude.

Summary statistics of the measured GE elevation errors are presented in Table 1. In general, the MAE of GE elevation is 10.72 meters, the measured RMSE for GE elevation is 22.31 meters. In the comparison to GPS benchmarks, GE elevation exhibits a BE95 of ±43.72 meters. Another important descriptor of vertical accuracy is the mean error, or bias, which indicates if the GE elevation has an overall vertical offset (either positive or negative) from the true ground level. In this assessment, the ME of GE elevation is 0.13 meters, indicating that GE does not have a significant bias. Fig 4 describes the error distribution pattern along the latitude and longitude in the conterminous USA, indicating that the GE elevation accuracy varies by the location.

**Table 1:** Error statistics of the accuracy assessment vs. GPS benchmarks.

Sample Size	20131
Min. AE(m)	0.00
Max. AE(m)	198.79
MAE(m)	10.72
ME(m)	0.13
Std. Dev.(m)	22.31
RMSE(m)	22.31
BE95(m)	±43.72

### GE vs. roadway monuments

**Fig 5:**
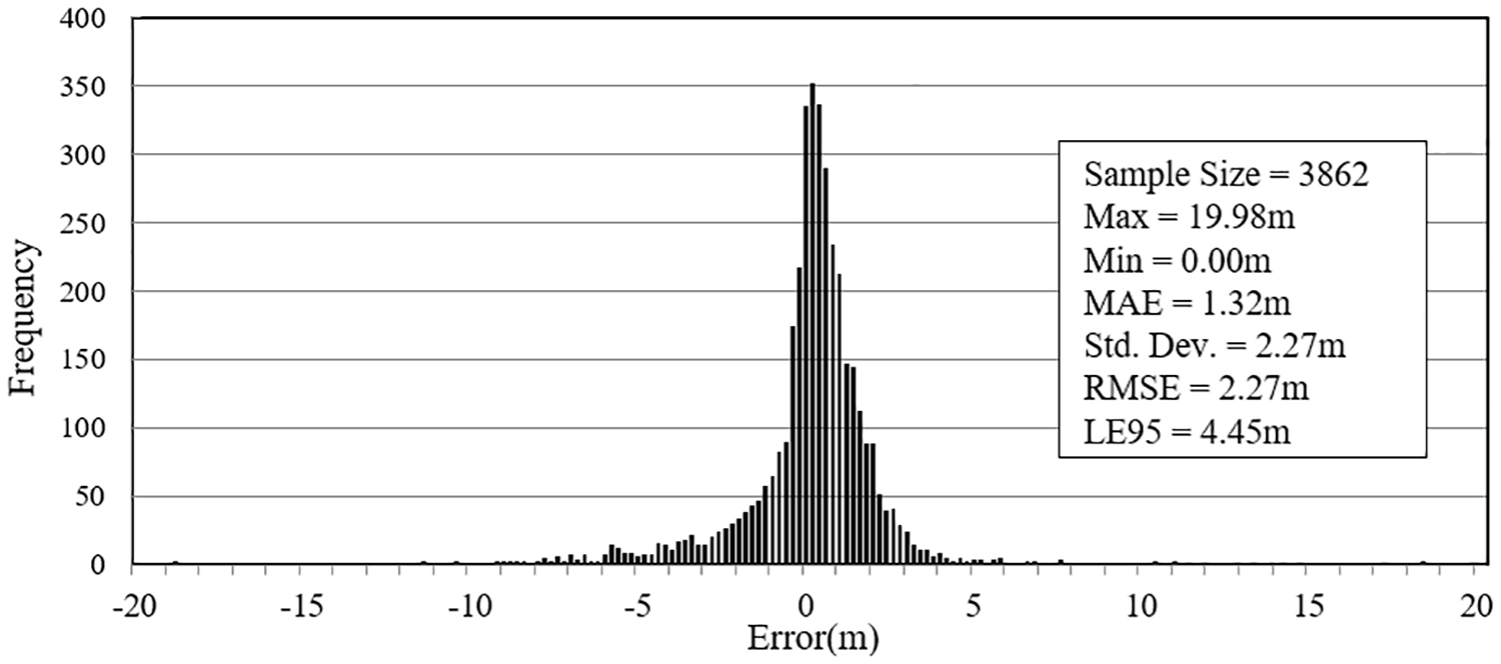
Frequency histogram of GE elevation error vs. roadway monuments.

The comparison above shows the GE elevation accuracy on a national wide scale. However, for transportation applications, the elevation accuracy along roadways is a more informative measure of how trustworthy GE elevation data is. To address this issue, this study compared the extracted GE elevation with the roadway monuments data from California (CA), New York (NY), Texas (TX), Washington (WA), Wyoming (WY) and Minnesota (MN) State. The roadway monuments data have the centimetre-level accuracy on both horizontal and vertical positions. The results shown in Fig 5 indicate that the MAE of GE elevation is 1.32 meters and the RMSE is 2.27 meters, which is a significant improvement over the GPS benchmarks comparison. The results obtained for each state shown in Table 2 indicate that the accuracy of GE elevation along the roadway does not vary significantly by location (states).

**Table 2:** Error statistics of the accuracy assessment vs. roadway monuments.

State	Sample Size	Min. AE(m)	Max. AE(m)	MAE(m)	Std. Dev.(m)	RMSE(m)	BE95(m)	Mann-Whitney U Test (p value)
CA	431	0	19.98	1.46	2.33	2.35	±4.56	0.94*
NY	214	0	13.33	1.67	2.43	2.57	±4.77	0.65
TX	576	0	18.22	1.12	2.09	2.14	±4.20	0.99
WA	1270	0	18.82	1.67	2.79	2.81	±5.46	0.81
WY	117	0.08	19.1	2.22	2.88	3.04	±5.64	0.93
MN	1254	0	18.26	0.88	1.36	1.39	±2.68	0.94
ALL	3862	0	19.98	1.32	2.27	2.27	±4.45	0.93

* P-value is larger than 0.05, and we fail to reject the null hypothesis that two elevation datasets are equal.

**Fig 6:**
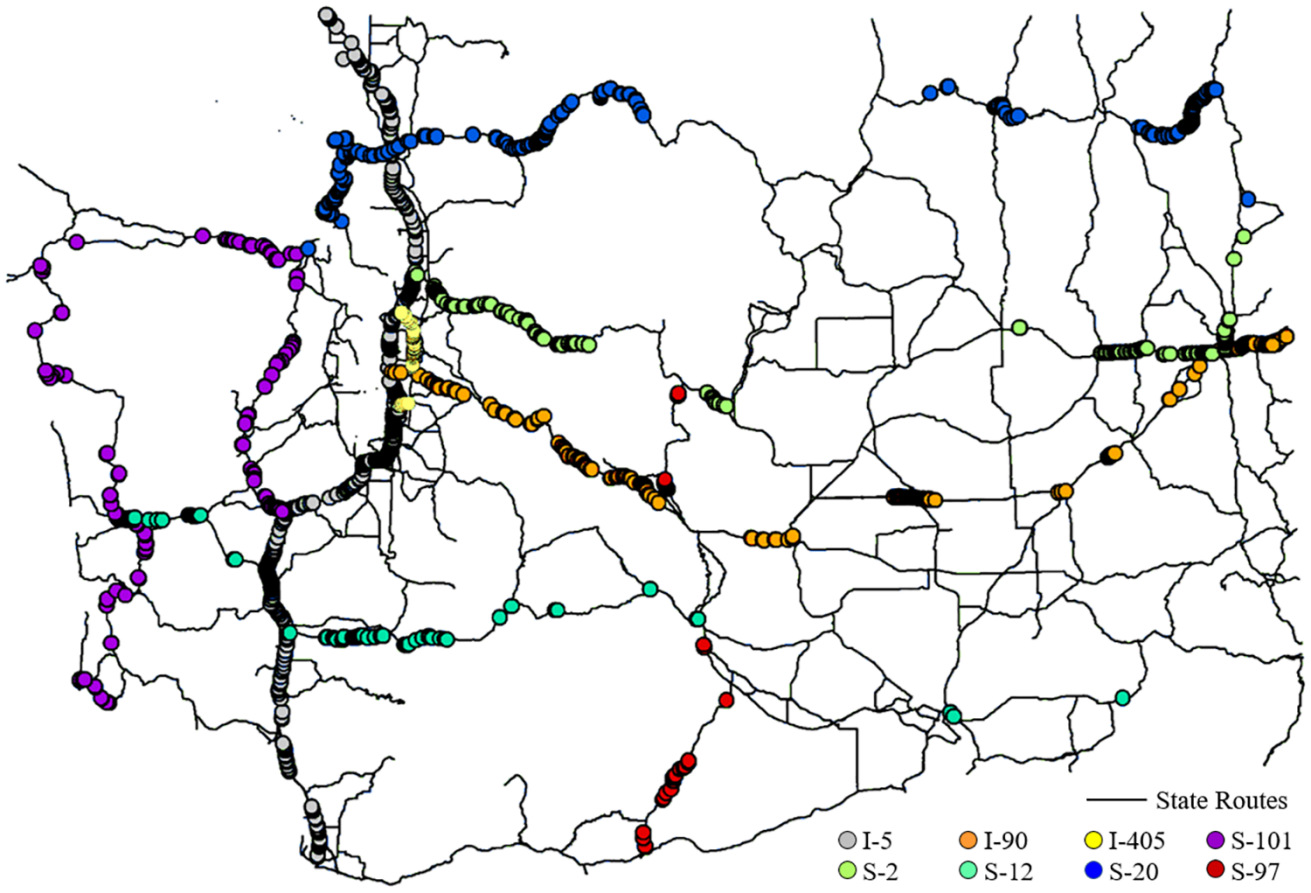
Sampling points distribution along roadways in Washington State.

To test whether GE elevation accuracy varies between different roadway types, this study explored the GE elevation error by looking into the roadway monuments data from Washington State categorized by the route code (see Fig 6). Three interstate freeways and five state highways are involved in this assessment. The results of each route shown in Table 3 indicate that the accuracy of GE elevation does not vary significantly between different routes and facility types.

**Table 3:** Error statistics of the accuracy assessment among different routes in Washington State.

Route	Sample Size	Min. AE(m)	Max. AE(m)	MAE(m)	Std. Dev.(m)	RMSE(m)	BE95(m)	Mann-Whitney U Test (p value)
I-5	443	0	11.34	1.65	2.77	2.89	±5.43	0.64*
I-405	43	0	13.52	1.44	2.72	2.74	±5.34	0.88
I-90	176	0.07	10.25	2.09	2.9	2.9	±5.68	0.94
S-101	138	0	18.82	1.28	3.04	3.07	±5.96	0.69
S-12	69	0.01	12.89	1.44	2.5	2.52	±4.90	0.92
S-2	137	0.02	10.23	2.08	2.81	2.85	±5.50	0.97
S-20	222	0	18.32	1.39	2.39	2.4	±4.69	0.99
S-97	42	0.01	11.44	2.15	2.93	3.34	±5.74	0.9
ALL	1270	0	18.82	1.67	2.79	2.81	±5.46	0.81

* P-value is larger than 0.05, and we fail to reject the null hypothesis that two elevation datasets are equal.

### GE vs. other digital elevation model (DEM)

**Fig 7:**
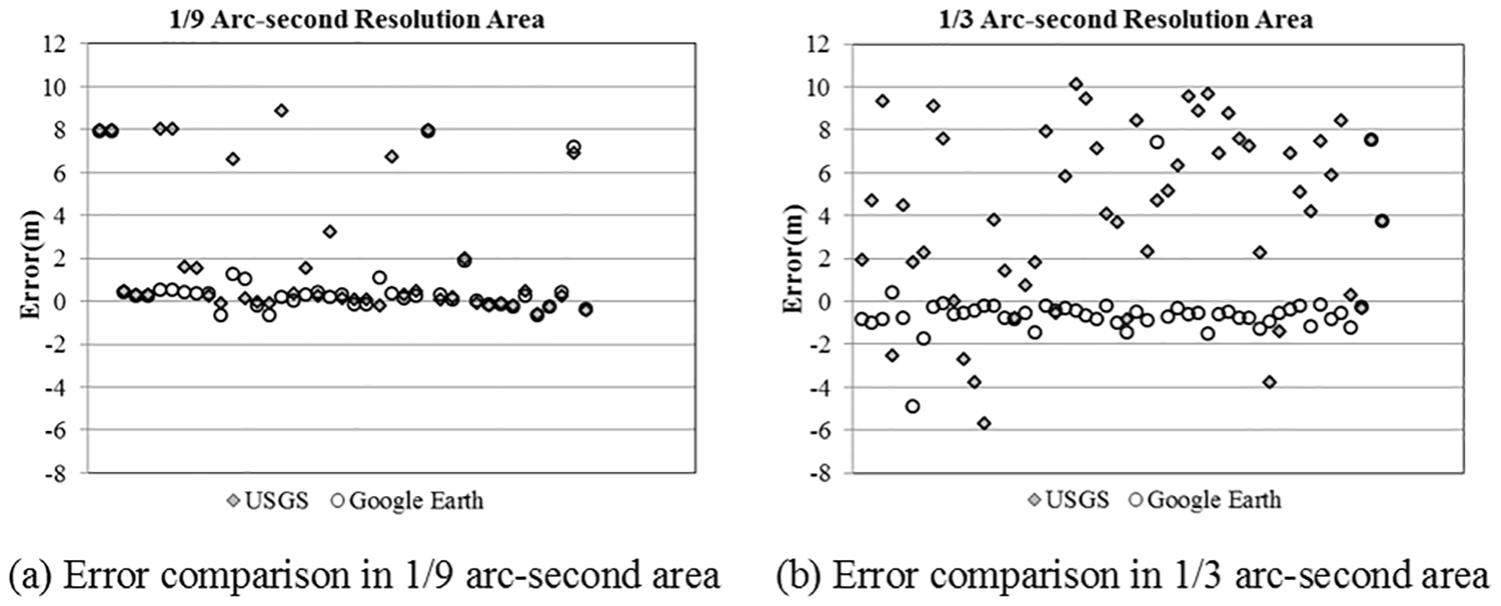
Error comparison between GE and USGS NED.

As mentioned in the introduction section, there are several other data sources providing global or regional DEMs. The USGS NED is one of the well-known DEMs covering the United States nationwide. The data in USGS NED are available at a grid spacing of 1 arc-second (about 30 meters) for the conterminous USA, and at 1/3 and 1/9 arc-second grids (approximately 10 and 3 meters, respectively) for parts of the nation. The research team conducted a local study that compared the elevation data extracted from Google Earth and USGS NED with the WSDOT monuments data (centimetre-level accuracy) along I-5 in Washington State. The comparison result shown in Fig 7(a) suggests that the elevation data extracted from Google Earth is at least as accurate as the 1/9 arc-second resolution USGS NED data, in which the mean error of GE elevation data is 0.97m smaller and the t-test statistic is 2.55; and Fig 7(b) suggests that the accuracy of the 1/3 arc-second resolution USGS NED data is significantly lower than that of the Google Earth elevation data, in which the mean error of GE elevation data is 3.84m smaller and the t-test statistic is 7.94. Based on this, Google Earth should be considered a valuable source of nationwide roadway elevation data, with coverage including the areas in which only 1/3 arc-second resolution data are available from USGS NED.

## Implementation and validation

Through the above analysis and tests, this study demonstrated that GE is a valuable elevation data source for transportation applications. This section implements and validates the extraction method developed in this study. Three scenarios are selected to test the performance of coordinates transformation method, multi-layered roadway recognition, and slope segmentation by grade calculations.

### Scenario 1: Extracting roadway elevation differentiating by directions

**Fig 8:**
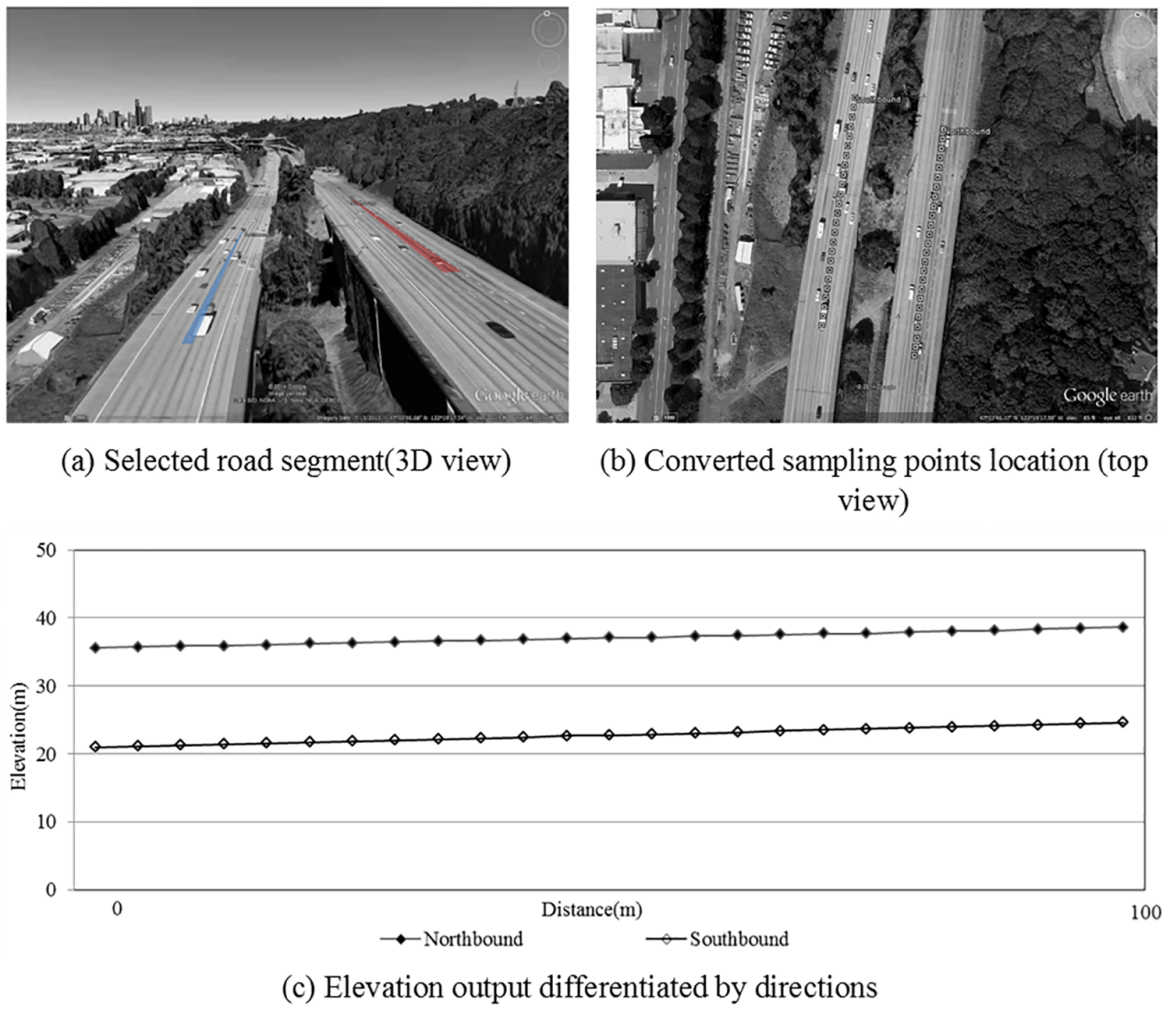
Extracting roadway elevation differentiating by directions.

Since the GE and general roadway GIS use different coordinates system, the performance of coordinate transformation method proposed in this study is essential to ensure the accuracy of extracted elevation results. For testing, a freeway segment with different subgrade elevation on the two directions is selected as shown in Fig 8(a). The longitude and latitude of the starting and ending points for each direction at the roadway centerline are input into the coordinate transformation method. Fig 8(b) shows the location of converted sampling points, which are appropriately located on the roadway centerline. By acquiring the elevation of converted sampling points through the GE API, the roadway surface elevation can be extracted as shown in Fig 8(c). This demonstrates that the proposed extraction method is capable of differentiating roadway surface elevation by direction, indicating that the extraction method can locate the extracting route accurately with GE.

For transportation applications, the precision of elevation data is another important factor in addition to the absolute accuracy. Even if the overall accuracy of elevation data source is low, the extracted data can still be used if the elevations of sampling points are precise to each other along a certain roadway segment. In this scenario, the extracted segments are both in the constant grade sections, thus the extracted elevations of the sampling points are compared with the elevation on the ideal straight-lines connecting the starting and ending points of these segments. The mean of the differences is 0.06m and 0.03m for the northbound and southbound respectively, indicating that the precision of GE elevation data along roadways is satisfactory.

### Scenario 2: Multi-layered roadway recognition in freeway segment

**Fig 9:**
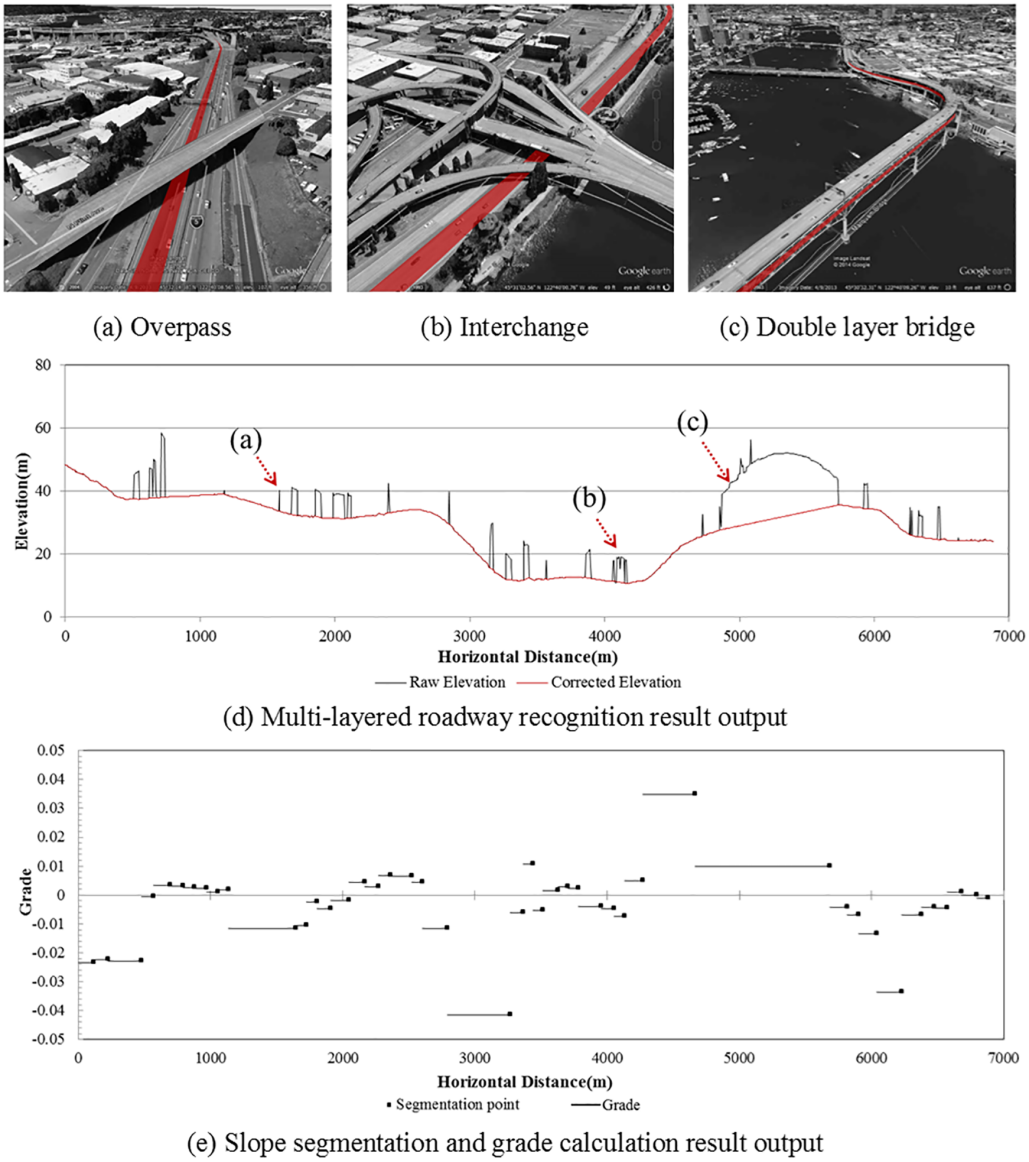
Multi-layered roadway recognition and slope segmentation along I-5 southbound in Portland, OR.

To test the performance of multi-layered roadway recognition method, we chose the Interstate Freeway No.5 southbound in the downtown area of Portland, OR, which contains 17 overlapping segments including interchanges and a double layer bridge. The horizontal distance between two successive sampling points (sampling resolution) is 5m, and the thresholds were set as follows: *α* = 1*m*, *β* = 5*m* and *N* = 5. The recognition results are shown in Fig 9. Fig 9(a) shows a situation where the desired roadway is covered by an overpass, marked as (a) in Fig 9(d). Fig 9(b) and 9(c) show cases with an interchange and double layer bridge respectively, which are marked as (b) and (c) in Fig 9(d) accordingly. From Fig 9(a) through 9(d), it is clear that all the overlapping segments were identified by the proposed methodology.

### Scenario 3: Slope segmentation and grade calculation in freeway segment

In this scenario, we chose the roadway segment described in Scenario 2 to test the performance of slope segmentation and grade calculation method. Based on the corrected elevation data, the extracted route can be divided into several segments by slope grade as shown in Fig 9(e). In Fig 9(e), it is clear that the locations of segmentation points are consistent with the roadway grade changes shown in Fig 9(d), indicating that the proposed segmentation method works well with the elevation data acquired from GE.

## Conclusion and future work

This paper aims at evaluating Google Earth as a possible elevation data source for transportation applications. A method for extracting roadway elevation data from GE was developed. The elevation extraction method includes GE viewbox parameters determination, Coordinate transformation, Multi-layered roadway recognition and data correction, and Slope segmentation and grade calculation. To understand the accuracy of GE elevation data, a comprehensive accuracy assessment on GE elevation data was conducted in the area of conterminous USA. First, the GE elevation data was compared with the ground truth data from nationwide GPS benchmarks and six states’ roadway monuments in the conterminous USA. Then, the accuracy assessment also compared GE elevation with the elevation raster data from USGS NED. Finally, the proposed extraction method was implemented and validated in three scenarios including (1) Extracting roadway elevation differentiating by directions, (2) Multi-layered roadway recognition in freeway segment and (3) Slope segmentation and grade calculation in freeway segment. The following conclusions can be drawn from the testing and validation results:

1. The accuracy of elevation data from GE is better along roadways compared to other elevation data sources in the conterminous USA, with MAE, RMSE, and GE roadway elevation error standard deviation of 1.32*m*,2.27*m* and 2.27*m* respectively;
2. Google Earth elevation data is a valuable resource for transportation applications. The precision of GE elevation data along roadways is satisfactory, and there is no evidence showing the accuracy of GE roadway elevation varies significantly between states or route types; and
3. The proposed extraction methods can locate the extracting route accurately, and can recognize multi-layered roadway section and segment the extracted route by grade automatically.

There are a few points deserve discussions. First, Google Earth records elevation information on the ground surface. Thus, under some circumstances, Google Earth elevation data may be inaccurate where a roadway is under an overhead structure or a roadway is within a tunnel. The roadway grade design guidelines from the American Association of State Highway and Transportation Officials’ (AASHTO) A Policy on Geometric Design of Highways and Streets [22] can be considered to identify the abnormal elevation values. Second, it is useful to develop some procedures to automatically correct the abnormal elevation data and ensure the elevation data quality from Google Earth. For example, a moving average method can be used to smooth the sudden spike and dip of curves. Third, for large-scale data extraction from Google Earth, it is necessary to design a roadway network geo-database and extract elevation information automatically[23].

Future research may continue our investigation along the following four directions: First, the extraction method itself can be improved by testing the accuracy of corrected multi-layered roadway elevation and the calculated grade, and by tuning of the thresholds used in the method to make them suitable for different roadway types. Second, applications of the GE elevation data and extraction method can be investigated such as testing elevation change effects on vehicle fuel efficiency, assessing the impacts of elevation change on congestion under shortest travel time and most energy efficient route choice alternatives, and exploring the influence of elevation change on the choice of non-motorized travel modes. Third, elevation data may be combined with the GIS data on an online and easy to access platform to support various transportation analyses on roadway networks. Fourth, it is useful to develop a data cleaning method (e.g., a moving average method) for obtaining more accurate roadway elevation data from Google Earth [24].

## Supporting information

S1 File Analysis data.zip contains data and analysis results for this study. (ZIP)
